# Pathways Linking Parental Support to Adolescents’ Reading Proficiency: A Social Cognitive Theory Perspective

**DOI:** 10.3389/fpsyg.2021.746608

**Published:** 2021-10-20

**Authors:** Xueliang Chen, Jie Hu

**Affiliations:** Department of Linguistics, Zhejiang University, Hangzhou, China

**Keywords:** perceived parental support, self-efficacy, mastery goal orientation, reading enjoyment, PISA reading

## Abstract

Parental support is essential to children’s motivation and academic functioning. However, few studies have investigated the pathways linking perceived parental support to children’s achievement in reading during adolescence. This study aims to fill this gap by systematically investigating the relationships among perceived support from parents, adolescents’ motivational beliefs, and reading proficiency based on Bandura’s social cognitive theory. A range of motivational processes are explored, including self-efficacy, goals, and values. Using the China sample from the Programme for International Student Assessment (PISA) 2018, which includes 12,058 adolescents from 361 schools, this study proposed two competing models based on different accounts of self-efficacy beliefs. Multilevel path analysis is adopted as the analytic method. The results suggest that perceived support from parents has a statistically significant but negligible relationship with adolescents’ reading proficiency. However, this relationship is mediated by nuanced pathways such as self-efficacy beliefs, mastery goal orientation, and reading enjoyment. Findings of this study provide evidence in support of the top-down theory of self-efficacy in the reading context and also contribute to a better understanding of the interactions between different motivational processes. Theoretical and practical implications of this study are discussed, and suggestions for future research are offered.

## Introduction

Attaining sufficient reading skills is a prerequisite for later academic performance and successful integration into society (OECD, 2019). Although many factors may affect children’s reading comprehension, family influences such as support from parents have been shown to explain a unique proportion of variance in reading proficiency (Klauda, 2009). Research conducted with children during early childhood provides ample evidence for the benefits of such support (Sénéchal et al., 1998; Inoue et al., 2018; Gao et al., 2021), and continued support during adolescence can also have a huge impact on children’s reading motivation and behaviors (Klauda, 2009).

However, studies have shown that compared with early childhood, support during adolescence is drastically reduced, with potentially deleterious effects on children’s growth (Jacobs et al., 2002; Wigfield et al., 2006; Klauda, 2009; Merga, 2018). There are several possible reasons that might account for this phenomenon. First, children tend to spend more time in formal learning contexts than in the home as they grow older. Therefore, opportunities for parental involvement might be limited. Second, many adolescents have generally developed greater autonomy and independence and may not necessarily welcome parental interference (Merga, 2018). Nonetheless, recent studies suggest that support from parents continues to produce benefits for children’s educational attainment in various domains during this developmental period (Zhan and Sherraden, 2011; Benner et al., 2016; Chen et al., 2019). How such support translates into adolescents’ progress, though, remains underexplored.

The goal of this study is to investigate the motivational pathways underlying the association between adolescents’ perceived support from parents and their achievement in reading. Bandura’s social cognitive theory of psychological functioning (Bandura, 1986, 1997, 2001) is adopted as the conceptual framework. This theory highlights the central role of the social context in human learning, which interacts with various personal (e.g., self-efficacy beliefs, goals, values, etc.) and behavioral factors (e.g., achievement) (Bandura, 1977, 2001). As such, it aligns closely with the scope of the present study.

## Parental Support and Children’s Reading Proficiency

As parents are often children’s first teachers, a cornucopia of studies has investigated the role of parents’ involvement in children’s learning in early childhood (e.g., Sénéchal et al., 1998; Inoue et al., 2018; Kim and Riley, 2021). Many of these studies have focused on the development of children’s language and emergent literacy skills. For example, shared book reading, as one of the most studied literacy practices that occurs in the home context, has been positively associated with a wide range of language and literacy measures, such as children’s receptive and expressive vocabulary (Sénéchal et al., 1998), reading accuracy and fluency (Inoue et al., 2018), and reading achievement (Kim and Riley, 2021). Furthermore, parents’ provision of learning resources at home (e.g., Gao et al., 2021) and frequent visits to the library (e.g., Katzir et al., 2009) have been identified as positive predictors of reading comprehension in primary school students. Apart from these deliberate literacy practices, parents can also unconsciously serve as role models for their children through their own reading emotions and behaviors. For example, parents who hold positive emotions about reading can pass on such emotions to their children and help them develop a love of reading, thereby contributing to their reading achievement (Nalipay et al., 2019). Similar intergenerational transfer also occurs when children imitate the reading habits of their parents in the home and read on their own without active parental involvement (Mancini et al., 2017). These findings are consistent with the early theoretical accounts of Bandura’s social cognitive theory (Bandura, 1977; Schunk and DiBenedetto, 2016, 2020), which claim that much human learning occurs by observing and modeling other people’s behaviors.

However, studies examining the role of parents in adolescents’ reading have been limited, presumably because parental involvement is less frequent or obvious during this developmental period (Merga, 2018). Nonetheless, the extant studies on this topic clearly support the benefits of continued parental support for adolescents’ reading proficiency (Song et al., 2015; Pfost et al., 2016). Using a combination of questionnaires and interviews, Pfost et al. (2016) found that there was a significant association between mothers’ reading attitudes and behaviors with those of their adolescent children. Based on their findings, they argued that even as children reach adolescence, the influence of parents continues to be evident in children’s reading. In a longitudinal study, Song et al. (2015) also demonstrated that perceived support from parents predicted adolescents’ academic achievement in English and other subjects across school years. These studies suggest that more research is needed to buttress the case for parental support during adolescence.

## Self-Efficacy Beliefs and Reading Proficiency

One of the central components of Bandura’s social cognitive theory is self-efficacy, which is defined as individuals’ perceived ability to perform at designated levels in a given domain (Bandura, 1997; Klassen and Usher, 2010). Individual self-efficacy beliefs are influenced by the social context, such as different forms of social persuasion and vicarious experiences (e.g., support from parents) (Schunk and Usher, 2019). It has been reported that students’ perceived support from parents and teachers is positively associated with their perceptions of competence and that such self-efficacy beliefs serve as explanatory mechanisms for achievement (Diaconu-Gherasim et al., 2020).

In accordance with Bandura’s domain-specific conceptualization of self-efficacy (Schunk and Usher, 2019), reading self-efficacy is defined as individuals’ self-perceived competence in the reading domain (Carroll and Fox, 2017). Many studies have supported a positive relationship between reading self-efficacy and reading proficiency (Smith et al., 2012; Peura et al., 2019a). These studies suggest that children with high perceptions about their competence to read tend to read on their own initiative and have more perseverance relative to children with low self-efficacy. In addition, children with high reading self-efficacy are more willing to select more challenging reading materials (Schiefele et al., 2012). However, inconsistent findings also exist. As some studies suggest, high reading self-efficacy does not necessarily predict high reading proficiency (Carroll and Fox, 2017). Individuals might have inflated perceptions of their real competence and stop working hard, which can negatively affect their achievement.

Although Bandura regarded self-efficacy as context-specific, he also conceded that self-efficacy beliefs might generalize beyond any given situation (Bandura, 1986). In relation to this question, scholars have probed into the directionality of the relationship between domain-general self-efficacy and domain-specific self-efficacy, where general self-efficacy is defined as individuals’ self-perceived ability to perform across a range of different situations (Judge and Kammeyer-Mueller, 2012). This line of work has resulted in two divergent perspectives: top-down theories and bottom-up theories (Grether et al., 2018). Bottom-up theories support the assertion that individual experiences in specific areas of life can add up to a general sense of competence (e.g., Shavelson et al., 1976). On the contrary, top-down theories advocate that domain-specific self-efficacy beliefs form as a result of more stable, long-term self-efficacy beliefs (e.g., Shelton, 1990). In a longitudinal study addressing this topic, Miyoshi (2012) found support for both bottom-up and top-down effects. Similar findings were also obtained by Grether et al. (2018), who investigated this issue using a cross-lagged design and found evidence in support of both theoretical accounts.

In the context of reading, studies have also shown that whether self-efficacy beliefs can predict reading proficiency depends on the specificity of self-efficacy (Peura et al., 2019a,b). For example, Peura et al. (2019a) showed that self-efficacy assessed at different levels are differentially associated with reading fluency. To further clarify such relationships, Peura et al. (2019b) went on to demonstrate that specific and intermediate self-efficacy are associated with reading fluency, but no such relationship exists between general self-efficacy and reading fluency. Overall, these studies suggest that research on this topic is still in an inchoate stage, and mixed empirical findings preclude definitive evidence in support of any one theory. Additional research is necessary to further address this issue.

## Mastery Goal Orientation, Reading Enjoyment and Reading Proficiency

Two other cognitive processes mentioned in Bandura’s framework are goals and values, which are defined as what people actively aim to attain (learning goal versus performance goal) and their subjective appraisals of task importance and utility, respectively (Schunk and Usher, 2019). These two constructs are also shaped by the social context and associated with various motivational processes. In this study, they are operationalized as mastery goal orientation and reading enjoyment.

Mastery goal orientation represents individuals’ mastery-approach orientation of achievement goals (Ryan and Deci, 2020). Although it was originally proposed in the achievement goal literature, it overlaps to a large extent with Bandura’s conceptualization of learning goals as well. Such goals are influenced by environmental stimuli and interact with contextual factors to have an effect on individuals’ motivation and achievement (Putwain et al., 2018). In a longitudinal study, Song et al. (2015) observed that the type of messages students receive from socially significant others is related to their adoption of different goals. When students perceive positive support from social agents, they are more likely to pursue goals aimed at knowledge acquisition rather than performance comparison, with different goals differentially related to achievement outcomes. In other words, mastery goals might serve as a mediator between social support and achievement. Students who have mastery goals are also considered intrinsically motivated and have high task enjoyment (Donovan et al., 2018). Such motivational processes have been positively associated with achievement outcomes in many domains (e.g., Huang, 2011; Putwain et al., 2018; Eccles and Wigfield, 2020), though less consistent findings have been observed for students’ reading outcomes (Wolters et al., 2017). Recent studies on reading indicate that potential mechanisms might exist that could explain the association between mastery goal orientation and students’ reading outcomes (Wolters et al., 2017; Cho et al., 2018, 2019).

Reading enjoyment represents the intrinsic value or enjoyment individuals derive out of reading tasks. Empirical evidence has consistently supported a positive association between reading enjoyment and reading proficiency (Schiefele et al., 2012; Nalipay et al., 2019). Using a secondary dataset, Rogiers et al. (2020) identified reading enjoyment as one of the key predictors of skilled readers among 15-year-old adolescents. Other studies have shown that individuals with high enjoyment of reading tend to be involved more frequently in reading activities, become more engaged in reading tasks, and even choose more challenging reading materials relative to those who do not enjoy reading (Schiefele et al., 2012). The social context also plays a role in shaping individual’s reading enjoyment. For example, many studies in early childhood education have documented a link between parental reading behaviors and children’s enjoyment of reading activities (e.g., Pfost et al., 2016; Preece and Levy, 2020). Research on adolescents also provides evidence that parents’ reading behaviors are closely related to adolescents’ reading enjoyment (Nalipay et al., 2019), and that reading enjoyment mediates the association between contextual support and reading achievement (Ma et al., 2021).

Taken together, these studies suggest that mastery goal orientation and reading enjoyment represent two motivational pathways that might account for variations in achievement outcomes. Both processes are shaped by the social context and also have a direct relationship with achievement. Mastery goal orientation also has a direct relationship with individuals’ task enjoyment.

## Materials and Methods

### Sample

Secondary data from the Programme for International Student Assessment (PISA) are used. PISA is an international large-scale assessment system that investigates 15-year-old adolescents’ performance in reading, mathematics, and science. Starting in 2020, it has been implemented every 3years in many parts of the world, informing national policy-making and serving as a benchmark for monitoring educational progress and making international comparisons. As of today, PISA has become the most authoritative student assessment system in the world. In 2018, reading is specified as the major domain in PISA. In addition to adolescents’ reading scores, a number of background indices pertaining to their motivation are also provided.

In this study, the China sample is extracted from the PISA dataset for use, which comprises 12,058 students from 361 schools. A two-stage stratified sampling approach was implemented to identify potential students from four provinces in China, including Beijing, Shanghai, Jiangsu and Zhejiang (B-S-J-Z). In the first stage, schools were systematically sampled from a comprehensive list of all PISA-eligible schools containing 15-year-old students. School characteristics such as geographical location and urbanization were also taken into account to improve the precision of selection. In the second stage, individual students (usually 35) were sampled from each of the sampled schools from a list of all 15-year-old students. To provide an objective measure for sampling coverage, different coverage indices (e.g., coverage rates of the national population, the estimated school population, the sampling frame, etc.) are calculated, which range from 0.81 to 1.00 on a 0–1 scale (OECD, 2021). During the administration of PISA tests, national project managers worked in compliance with the testing procedures stipulated by international contractors, and in collaboration with assistants and school-level staff. The Chinese students who are represented in the ultimate PISA dataset range from 8^th^ to 12^th^ grade and have a mean age of 15.773years with a standard deviation of 0.293. Students also come from diverse socioeconomic backgrounds according to a composite socioeconomic index provided in the PISA dataset (*M*=−0.362, SD=1.087). Student representation by gender is also balanced, with about half of the students being female (*N*=5,775, ratio: 47.89%).

### Measures

A total of 11 variables are selected from the PISA 2018 dataset in accordance with the hypothesized model, which include six variables of substantive interest and five control variables.

### Perceived Parental Support

This variable refers to adolescents’ perceived support from their parents. Adolescents expressed their agreement or disagreement with three questionnaire items (e.g., “My parents support my educational efforts and achievements.”) on a Likert scale with four response categories, ranging from “strongly disagree” “disagree” to “agree” and “strongly agree.” Higher scores on this scale represent higher perceived support from parents. As a measure of internal reliability, coefficient omega is reported instead of Cronbach’s alpha because the tau-equivalence assumption of the latter index is mostly untenable in real-life situations (McNeish, 2017). The omega value for parental support is 0.910 with a 95% confidence interval (CI) of [0.905, 0.915], which indicates a narrow interval range.

### Self-Efficacy Beliefs

#### General Self-Efficacy

This variable refers to adolescents’ confidence in themselves and their ability to deal with difficult situations. It is measured with five items (e.g., “I usually manage one way or another.”) on a four-point Likert scale with four response categories, ranging from “strongly disagree” to “strongly agree.” Higher scores represent higher self-efficacy. Coefficient omega for this scale is 0.807, and the 95% CI is [0.800, 0.815].

#### Reading Self-Efficacy

This variable refers to adolescents’ self-perceived competence in the reading domain. It is measured on a four-point Likert scale with three items (e.g., “I am able to understand difficult texts.”). The four response categories range from “strongly disagree” to “strongly agree.” Higher scores represent higher reading self-efficacy. Coefficient omega for this scale is 0.803, and the 95% CI is [0.795, 0.810].

### Mastery Goal Orientation

This variable refers to the mastery-approach orientation of adolescents’ achievement goals, in other words, their intention to acquire competence through the mastery of the subject matter in a given domain. Three items (e.g., “My goal is to learn as much as possible.”) are used to measure this construct on a five-point Likert scale that ranges from “not at all true of me,” “slightly true of me,” “moderately true of me,” to “very true of me,” and “extremely true of me.” Higher scores represent higher mastery goal orientation. Coefficient omega for this scale is 0.763, and the 95% CI is [0.755,0.771].

### Reading Enjoyment

This variable refers to adolescents’ enjoyment of reading. It is measured with five items (e.g., “Reading is one of my favourite hobbies.”) on a Likert scale with four response categories, ranging from “strongly disagree” to “strongly agree.” Since some items are negatively worded in this scale, they are reverse coded before the main analysis. Higher scores on the scale represent higher enjoyment of reading. Coefficient omega for this scale is 0.811, and the 95% CI is [0.805,0.818].

In the PISA dataset, a composite index is provided for each of the afore-mentioned constructs, which has been scaled using Item Response Theory (IRT) techniques. Contrary to the traditional practice of summing item scores as a single value, the IRT techniques calibrate/estimate the latent constructs measured by the items based different probabilistic models, which assume a nonlinear relationship between item responses and latent constructs. This approach has been gaining popularity and is considered as a far superior alternative with greater measurement precision (OECD, 2009). Detailed questionnaire items used for measuring these variables are listed in the Appendix A.

### Reading Proficiency

The definition of reading proficiency in PISA 2018 is given as “understanding, using, evaluating, reflecting on and engaging with texts in order to achieve one’s goals, to develop one’s knowledge and potential and to participate in society” (OECD, 2019, p.28). To accurately measure this construct, the PISA authorities manipulated such factors as text type, text difficulty, domain coverage, response format, etc., to produce a large pool of reading items. A matrix sampling design was implemented for the actual testing, with items grouped into different booklets and each booklet containing different items. Different students were given different booklets for assessing their reading proficiency. Because different items were used for different students, PISA used IRT techniques to calibrate item-invariant person estimates (e.g., reading proficiency estimates). To be specific, the two-parameter logistic model was used for dichotomous items, while the generalized partial credit model was used for polytomous items. Instead of providing a single point estimate, the corresponding IRT models produced a posterior distribution of student abilities, from which ten values are randomly drawn as indicators of adolescents’ reading proficiency. These values are called plausible values. It is recommended that all the plausible values should be used in the data analysis (OECD, 2009).

### Control Variables

A total of five variables are used in this study as control variables, which include student gender, adolescents’ socioeconomic status (ESCS), school type, school size, and student–teacher ratio. The selection of such variables is justified by empirical studies showing their significance, which will be elaborated below.

Student gender and school type are both measured with a single questionnaire item, each with only two categories. Dummy coding is used to recode these variables (Gender: male=0, female=1; School type: private=0, public=1). ESCS is derived from a combination of three variables, including home possessions, parents’ highest occupational status, and parents’ highest level of education. Scores on these variables are used to provide a composite measure of students’ socioeconomic status in the PISA dataset. School size is the total number of student enrollment at a given school, while student–teacher ratio is calculated as the proportion of total students by the total number of teachers. Technical details regarding the calculation and construction of these indices can be found in the PISA 2018 technical report (OECD, 2021).

Demographic variables such as gender and ESCS have been consistently shown to affect students’ reading proficiency (Boerma et al., 2017; Cho et al., 2018; Ma et al., 2021), and therefore, they are included as student-level controls. Previous studies have also suggested that school-level factors such as school type, size, and student–teacher ratio have a significant effect on students’ reading outcomes (e.g., Lim and Jung, 2019; Xiao et al., 2019). Since this study aims to investigate the unique role of student-level factors, school-level factors are also controlled to partial out their effects. Descriptive statistics of all the variables are provided in Table 1. Bivariate correlations of the variables are provided in in the Appendix B.

**Table 1:** Descriptive statistics of the variables.

Measures	Mean	SD	Minimum	Maximum
Support	0.003	0.926	−2.450	1.030
G-Efficacy	−0.075	0.955	−3.170	2.370
Mastery	0.061	0.907	−2.530	1.850
R-Efficacy	0.079	0.862	−2.440	1.880
Enjoyment	0.981	0.844	−2.710	2.660
Read*	560.515	89.933	172.490	878.240
Gender	0.480	0.500	0.000	1.000
ESCS	−0.362	1.084	−5.080	3.100
Type	0.860	0.344	0.000	1.000
Size	1883.752	1451.472	78.000	1340.000
Ratio	1.650	6.159	1.000	10.000

Support, perceived support from parents; G-efficacy, general self-efficacy; Mastery, mastery goal orientation; R-Efficacy, reading self-efficacy; Enjoyment, reading enjoyment; Read, reading proficiency. Gender, student gender; ESCS, socioeconomic status; Type, school type; Size: school size; Ratio, student–teacher ratio.

* The descriptive statistics for “Read” are averaged across the ten plausible values.

### Hypothesized Models

Two hypothesized models are proposed to test the plausibility of different motivational pathways underlying the association between perceived parental support and adolescents’ reading proficiency. As the foregoing literature review suggests, social support has been directly associated with self-efficacy beliefs, mastery goals and task values (Nalipay et al., 2019; Chen and Hu, 2020), and achievement outcomes. As a result, a direct relationship is hypothesized in this study between perceived support from parents and all motivational variables and reading proficiency. The two models differ only in the relationship between domain-general self-efficacy and domain-specific self-efficacy, with opposite directions specified in each model.

Model_1_ hypothesizes that perceived support from parents operates through three separate pathways to reading achievement. One is a serial pathway from general self-efficacy to reading self-efficacy, consistent with the top-down account of self-efficacy beliefs (Shelton, 1990). Both forms of self-efficacy are directly related to reading proficiency. A second pathway is from mastery goal orientation to reading enjoyment, both of which are also directly related to reading proficiency. A third pathway is from reading self-efficacy to adolescents’ reading enjoyment, consistent with previous studies showing a positive association between self-efficacy and task value appraisals within the control-value theory framework (Chapman et al., 2000; Pekrun, 2006; Ma et al., 2021).

**Figure 1:**
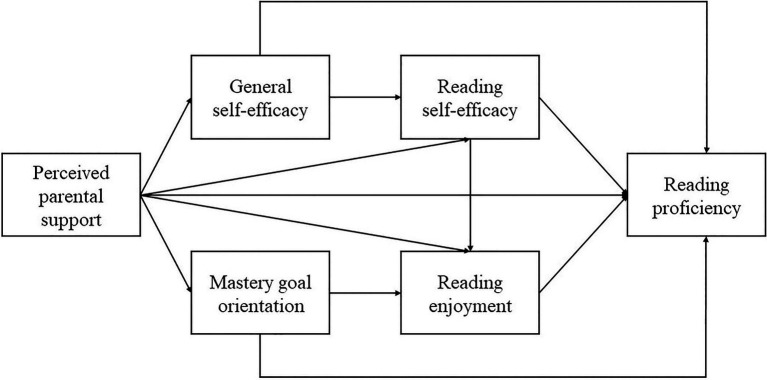
Hypothesized model (Model_1_) consistent with top-down theories.

**Figure 2:**
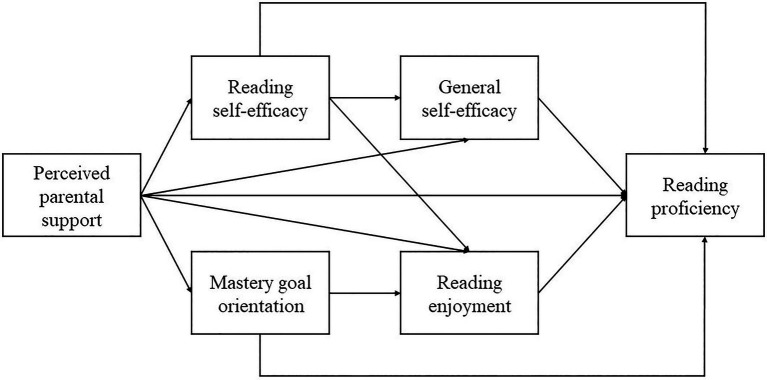
Hypothesized model (Model_2_) consistent with bottom-up theories.

Model_2_ retains most of the hypothesized pathways but reverses the direction between domain-general and domain-specific self-efficacy, consistent with a bottom-up processing mechanism of self-efficacy beliefs. In other words, it is postulated that perceived support from parents increases adolescents’ reading self-efficacy and then their general self-efficacy. Diagrams of these two hypothesized models are represented in Figures 1, 2.

### Analytic Method

This study adopts multilevel path analysis as the analytic method. Multilevel path analysis can be seen as a constrained version of the multilevel structural equation modeling framework, which combines the advantages of hierarchical linear modeling and structural equation modeling. As such, it can not only accommodate hierarchical data commonly seen in educational settings, but also investigate complex relationships among a set of variables simultaneously (Kline, 2015).

In this study, multilevel path analysis was performed using the *lavaan* package (Rosseel, 2012) in the programming environment R (R Core Team, 2020). A random intercept model was specified, and implicit model-based group mean centering was performed by default. Maximum likelihood estimation with robust standard errors (MLR) was used as the estimation method. Robust standard errors were calculated using the sandwich estimator (White, 1980).

Prior to the main analysis, an inspection of missing data revealed that missing data accounted for approximately 3% of the entire dataset. A Chi-square test of the missing mechanism showed that the data are missing completely at random (*p*>0.05). As a result, the expectation–maximization (EM) algorithm was used to impute the missing values of all the variables. All the variables were transformed to standardized *z*-scores with a mean of 0 and a standard deviation of 1, with the exception of student gender and school type. During the analysis, student weights were applied to reflect the design features of PISA’s two-stage stratified sampling approach (OECD, 2009). In addition, as there are ten plausible values used for students’ reading performance in PISA, ten analyses were performed separately following the same procedures.

## Results

### Variance Components and Intraclass Correlation Coefficients

Since PISA data are hierarchical in nature, with students nested in schools and schools nested in countries, this data structure needs to be statistically accounted for. Therefore, a null model with no predictor variable is specified for each of the main variables, which decomposes their total variance into between-school variance and within-school variance. An intraclass correlation coefficient is calculated using the following formula (Snijders and Bosker, 2012):

$$\mathrm{ICC}=\frac{\tau^{2}}{\tau^{2}+\sigma^{2}}$$ (1)

where $\tau^{2}\;$ represents the between-school variance; $\sigma^{2}\;$ represents the within-school variance. The ICC value represents the proportion of the total variance in a variable that is accounted for by the clustering of students within schools. It is recommended that when the ICC is greater than 0.1, a multilevel model should be preferred (Zhang and Wang, 2017).

As Table 2 suggests, although the ICC values for the main variables are mostly smaller than 0.1, adolescent students’ reading proficiency varies significantly across schools with an ICC of 0.466. Since school-level control variables are also introduced in this study, a multilevel model is appropriate.

**Table 2:** Variance components and intraclass correlation (ICC).

	*τ*^2^	*σ*^2^	ICC
Support	0.042	0.958	0.042
G-Efficacy	0.052	0.948	0.052
Mastery	0.051	0.949	0.051
R-Efficacy	0.087	0.913	0.087
Enjoyment	0.077	0.923	0.077
Read^*^	0.471	0.539	0.466

*τ*^2^, between-school variance; *σ*^2^, within-school variance. Support, perceived support from parents; G-efficacy, general self-efficacy; Mastery, mastery goal orientation; R-Efficacy, reading self-efficacy; Enjoyment, reading enjoyment; Read, reading proficiency.

* The variance components and ICC for “Read” are averaged across ten plausible values of adolescents’ reading proficiency.

### Evaluating the Fit of the Hypothesized Models

To evaluate the fit of the hypothesized models, some commonly used fit indices are reported, including Chi-square statistic (*χ*^2^) and its degrees of freedom (df), comparative fit index (CFI), Tucker–Lewis index (TLI), root mean square error of approximation (RMSEA), and standardized root mean square residual (SRMR). Kline (2015) provide general guidelines on the cutoff values for these fit indices, which are also applicable to the multilevel context. As a rule of thumb, the *χ*^2^/df statistic should generally be smaller than 3. CFI and TLI greater than 0.90 are considered acceptable, while RMSEA and SRMR smaller than 0.08 are adequate. In addition, since this study uses multilevel path analysis, two SRMR values are reported, one for the student level (SRMR*_w_*) and one for the school level (SRMR*_b_*). For each hypothesized model, the results are reported for all ten plausible values of adolescents’ reading proficiency.

As Table 3 suggests, Model_1_ shows acceptable fit with the data according to most fit indices. However, the *χ*^2^/df values are not acceptable for any of the plausible values. Since *χ*^2^/df is known to be sensitive to sample size (Kline, 2015), it may not be reliable for evaluating model fit in this study (*N*=12,058). Closer inspection reveals that one path in the model is insignificant, namely, the relationship between reading self-efficacy and reading proficiency. Therefore, it is decided that this insignificant path be removed before further analysis.

**Table 3:** Fit indices for Model_1_.

Model fit	*χ*^2^	df	CFI	TLI	RMSEA (90% CI)	SRMR*_b_*	SRMR*_w_*
PV1READ	89.740	2	0.994	0.915	0.060 (0.050,0.071)	0.000	0.013
PV2READ	89.740	2	0.994	0.916	0.060 (0.050,0.071)	0.000	0.013
PV3READ	89.740	2	0.994	0.915	0.060 (0.050,0.071)	0.000	0.013
PV4READ	89.740	2	0.994	0.916	0.060 (0.050,0.071)	0.000	0.013
PV5READ	89.740	2	0.994	0.916	0.060 (0.050,0.071)	0.000	0.013
PV6READ	89.740	2	0.994	0.915	0.060 (0.050,0.071)	0.000	0.013
PV7READ	89.740	2	0.994	0.916	0.060 (0.050,0.071)	0.000	0.013
PV8READ	89.740	2	0.994	0.916	0.060 (0.050,0.071)	0.000	0.013
PV9READ	89.740	2	0.994	0.916	0.060 (0.050,0.071)	0.000	0.013
PV10READ	89.740	2	0.994	0.916	0.060 (0.050,0.071)	0.000	0.013

χ2, Chi-square statistic; df, degree of freedom; CFI, comparative fit index; TLI, Tucker–Lewis index; RMSEA, root mean square error of approximation; SRMR_b_, standardized root mean square residual at the school level; SRMR_w_, standardized root mean square residual at the student level.

Results in Table 4 suggest that Model_2_ does not fit the data well. Even though the CFI and SRMR values are in the acceptable range, the other fit indices demonstrate that some serious misspecifications might have occurred. Since the relationship between reading self-efficacy and reading enjoyment in this model is also insignificant, this path will be removed to investigate whether removal of this path can lead to better fit.

**Table 4:** Fit indices for Model_2_.

Model fit	*χ*^2^	df	CFI	TLI	RMSEA (90% CI)	SRMR*_b_*	SRMR*_w_*
PV1READ	437.856	2	0.970	0.580	0.134 (0.124,0.145)	0.000	0.034
PV2READ	437.856	2	0.970	0.580	0.134 (0.124,0.145)	0.000	0.034
PV3READ	437.856	2	0.970	0.580	0.134 (0.124,0.145)	0.000	0.034
PV4READ	437.856	2	0.970	0.581	0.134 (0.124,0.145)	0.000	0.034
PV5READ	437.856	2	0.970	0.582	0.134 (0.124,0.145)	0.000	0.034
PV6READ	437.856	2	0.970	0.582	0.134 (0.124,0.145)	0.000	0.034
PV7READ	437.856	2	0.970	0.582	0.134 (0.124,0.145)	0.000	0.034
PV8READ	437.856	2	0.970	0.582	0.134 (0.124,0.145)	0.000	0.034
PV9READ	437.856	2	0.970	0.582	0.134 (0.124,0.145)	0.000	0.034
PV10READ	437.856	2	0.970	0.581	0.134 (0.124,0.145)	0.000	0.034

χ2, Chi-square statistic; df, degree of freedom; CFI, comparative fit index; TLI, Tucker–Lewis index; RMSEA, root mean square error of approximation; SRMR_b_, standardized root mean square residual at the school level; SRMR_w_, standardized root mean square residual at the student level.

### Fit Indices of the Revised Models

After the insignificant path has been removed, the fit of the revised models is evaluated using the same indices reported above. To facilitate reporting, the revised versions of Model_1_ and Model_2_ are renamed Model_3_ and Model_4_, respectively.

Results in Table 5 show that Model_3_ shows improvement in most of the indices, as evidenced by the greater TLI values and smaller RMSEA values. Given that the original model also fits the data well, a Chi-square difference test is performed to compare the fit of these two models. Ten comparisons were performed for each of the ten plausible values. Both significant and non-significant results were obtained. Despite the mixed evidence, the discrepancies between the two models suggest only a trivial difference in the results. For the sake of simplicity, the more parsimonious model was retained. Therefore, the revised model is accepted as the finalized model.

**Table 5:** Fit indices for Model_3_.

Model fit	*χ*^2^	df	CFI	TLI	RMSEA (90% CI)	SRMR*_b_*	SRMR*_w_*
PV1READ	91.940	3	0.994	0.943	0.050 (0.041,0.059)	0.000	0.013
PV2READ	94.366	3	0.994	0.941	0.050 (0.042,0.059)	0.000	0.013
PV3READ	96.222	3	0.994	0.940	0.051 (0.042,0.060)	0.000	0.013
PV4READ	96.404	3	0.994	0.940	0.051 (0.042,0.060)	0.000	0.013
PV5READ	101.181	3	0.993	0.937	0.052 (0.044,0.061)	0.000	0.014
PV6READ	100.214	3	0.993	0.937	0.052 (0.043,0.061)	0.000	0.014
PV7READ	102.098	3	0.993	0.937	0.052 (0.044,0.061)	0.000	0.014
PV8READ	99.921	3	0.993	0.938	0.052 (0.043,0.061)	0.000	0.014
PV9READ	92.770	3	0.994	0.943	0.050 (0.041,0.059)	0.000	0.013
PV10READ	92.237	3	0.994	0.943	0.050 (0.041,0.059)	0.000	0.013

χ2, Chi-square statistic; df, degree of freedom; CFI, comparative fit index; TLI, Tucker–Lewis index; RMSEA, root mean square error of approximation; SRMR_b_, standardized root mean square residual at the school level; SRMR_w_, standardized root mean square residual at the student level.

For Model_4_, however, model-data fit is poor even after removing the insignificant path, as indicated in Table 6 by the TLI and RMSEA values. As a result, Model_4_ is rejected and not considered for subsequent analysis.

**Table 6:** Fit indices for Model_4_.

Model fit	*χ*^2^	df	CFI	TLI	RMSEA (90% CI)	SRMR*_b_*	SRMR*_w_*
PV1READ	440.057	3	0.970	0.719	0.110 (0.101,0.119)	0.000	0.034
PV2READ	442.482	3	0.970	0.719	0.110 (0.102,0.119)	0.000	0.034
PV3READ	444.339	3	0.970	0.719	0.110 (0.102,0.119)	0.000	0.034
PV4READ	444.521	3	0.970	0.717	0.110 (0.102,0.119)	0.000	0.034
PV5READ	449.298	3	0.969	0.714	0.110 (0.103,0.119)	0.000	0.034
PV6READ	448.331	3	0.969	0.714	0.110 (0.102,0.120)	0.000	0.034
PV7READ	450.215	3	0.969	0.714	0.110 (0.103,0.119)	0.000	0.034
PV8READ	448.038	3	0.970	0.716	0.111 (0.102,0.120)	0.000	0.034
PV9READ	440.887	3	0.970	0.720	0.110 (0.101,0.119)	0.000	0.034
PV10READ	440.353	3	0.970	0.719	0.110 (0.101,0.119)	0.000	0.034

χ2, Chi-square statistic; df, degree of freedom; CFI, comparative fit index; TLI, Tucker–Lewis index; RMSEA, root mean square error of approximation; SRMR_b_, standardized root mean square residual at the school level; SRMR_w_, standardized root mean square residual at the student level.

### Parameter Estimates of the Finalized Model

After the finalized model has been established, the parameter estimates, standard errors, and *R*^2^ for the main variables are presented in Table 7. Due to space limits, effects of the control variables are provided in in the Appendix C. As Table 7 demonstrates, perceived support from parents has a significant but negligible relationship with adolescents’ reading enjoyment (*β*=0.095) and reading proficiency (*β*=0.047). However, a moderate association is found between perceived support and adolescents’ general self-efficacy (*β*=0.294) and mastery goal orientation (*β*=0.274), and between general self-efficacy and reading self-efficacy (*β*=0.393). The coefficient for mastery goal orientation in relation to reading enjoyment (*β*=0.087) is significant but small. On the other hand, enjoyment of reading is a positive predictor of adolescents’ reading proficiency (*β*=0.180). The proportion of variance explained for the endogenous variables is 0.132, 0.100, 0.251, 0.389, 0.173, respectively.

**Table 7:** Parameter estimates of the finalized model.

	G-Efficacy	Mastery	R-Efficacy	Enjoyment	Read
*Fixed effects*					
Support	0.294 (0.011)	0.274 (0.011)	0.074 (0.009)	0.095 (0.008)	0.047 (0.008)
G-Efficacy			0.393 (0.011)		−0.074 (0.008)
Mastery				0.064 (0.009)	0.027 (0.008)
R-Efficacy				0.550 (0.010)	
Enjoyment					0.180 (0.007)
*Random effects*					
*σ*^2^	0.868	0.900	0.749	0.611	0.496
*τ*^2^					0.331
*R*^2^	0.132	0.100	0.251	389	0.173

*σ*^2^, residual within-school variance; *τ*^2^, residual between-school variance. Since model-based group mean centering is used, between-school variances for some student-level variables are eliminated. Standard errors of the parameter estimates are listed in parenthesis. All the effects are statistically significant at the 0.001 level. Asterisks are omitted to save space.

## Discussion

As a core component of children’s support network during adolescence, parents play an indispensable role in adolescents’ life in the home and at school. However, studies suggest that parental support decreases during this period with potentially detrimental effects on adolescents’ motivation and academic outcomes (Jacobs et al., 2002; Wigfield et al., 2006; Klauda, 2009). This study investigates the relationship between perceived support from parents and adolescents’ reading proficiency within Bandura’s social cognitive theory. Potential pathways underlying this relationship are explored, which involve multiple motivational processes.

## The Role of Perceived Parental Support in Adolescents’ Reading

According to Bandura’ social cognitive theory, individual learning takes place in a social context and is shaped by various contextual factors, such as significant others like parents, teachers, etc. (Schunk and Usher, 2019). Although research suggests that support from parents has a strong association with children’s language and literacy-related skills during early childhood (e.g., Sénéchal et al., 1998; Inoue et al., 2018; Kim and Riley, 2021), this study found only a negligible albeit statistically significant relationship between perceived support from parents and reading proficiency among adolescents.

There might be two substantive explanations for this finding. First, children in early childhood are exposed more to family influences than adolescents since they spend more time in the home context. Parents are also more actively engaged in their learning during this particular developmental period by adopting different literacy practices, such as book reading (e.g., Rodriguez and Tamis-LeMonda, 2011), explicit teaching of alphabetical knowledge and vocabulary (e.g., Li et al., 2020), etc. As children start formal schooling, parents begin to assume reduced responsibilities and the mantle of teaching is transferred to teachers in formal learning contexts (Merga, 2018). Second, perceived parental support is measured in this study as a general construct, which does not pertain to any particular achievement setting. As such, it might not be directly associated with adolescents’ domain-specific achievement outcomes, such as reading proficiency. Instead, adolescents benefit from such support indirectly through their interpretations of the messages they receive, by altering their self-efficacy beliefs, goals and values, etc. (Klassen and Usher, 2010). The results in this study suggest multiple pathways that might be at work in the process, such as the path from general self-efficacy to reading self-efficacy, the path from mastery goal orientation to reading enjoyment, etc. Indeed, accumulating evidence supports these disparate pathways. For example, it has been shown that forms of verbal persuasion represent one critical source of self-efficacy beliefs (Bandura, 1977, 1986, 1997; Schunk and Usher, 2019; Ma et al., 2021). Also, when students receive positive feedback from socially significant others such as parents, they are more likely to adopt mastery goals rather than performance goals, which in turn affect their achievement (Song et al., 2015).

Methodologically, it is plausible to argue that the despite the high reliability of perceived parental support, the use of only three items to measure this construct makes it difficult to fully capture variations in adolescents’ self-reports. Since secondary data are used in this study, this methodological concern cannot be directly resolved. Future studies might use more observed items to measure this construct.

## The Mediation of Self-Efficacy Beliefs At Different Levels of Specificity

Self-efficacy is at the core of Bandura’ social cognitive theory. Although previous studies have established the mediational role of this construct between social support and achievement outcomes (Song et al., 2015), few studies have investigated the directionality of general and domain-specific self-efficacy beliefs and their differential effects on achievement (e.g., Peura et al., 2019a,b).

The findings in this study are consistent with a top-down theoretical account of self-efficacy beliefs, which claims that domains-specific perceptions of competence form as a result of more stable, long-term perceptions of competence (Shelton, 1990). Endorsing this perspective, Shelton (1990) claimed that only when general self-efficacy beliefs have become established can they direct behavioral outcomes and perceptions in specific domains. Using a cross-lagged design, Miyoshi (2012) found evidence that reciprocal relationships exist between these two types of constructs, but a top-down effect of general self-efficacy on domain-specific self-efficacy was also observed. Aiming to further clarify this issue, Grether et al. (2018) carried out a longitudinal study in both occupational and familial settings. Based on their results, they concluded that both reciprocal relations and a top-down effect exist. The current study investigates this issue in the reading context and within a broad framework examining the mediational role of different motivational pathways. While no causal statements can be made based on correlational data, this study nonetheless shows that a motivational pathway from general self-efficacy to reading self-efficacy is more appropriate for explaining the association between perceived parental support and adolescents’ reading proficiency.

On the contrary, the bottom-up theory of self-efficacy is not supported in this study, as shown by the poor fit indices of Model_2_ and Model_4_. This finding dovetails partially with those of previous longitudinal studies (Miyoshi, 2012; Grether et al., 2018), which present mixed evidence regarding the directionality of the relationship. The confusion emanates partly from the conceptualization of general self-efficacy (Grether et al., 2018). As Bandura (1997) stated, it is unclear what exactly a general self-efficacy scale measures because individuals might have different interpretations of the items in different contexts. In line with this thinking, this study hypothesizes that since general self-efficacy is measured as a domain-general construct, adolescents might draw on a range of experiences when answering the questionnaire items, including their belief of their reading competence. However, when reading self-efficacy is assessed, the questionnaire items only tap into adolescents’ perceptions of their reading competence, without any reference to their perceived competence in other domains. This might explain why the bottom-up relationship is not supported in this study. Nonetheless, it must be noted that the poor fit indices suggest only that the proposed network of relationships is not plausible. They do not refute the possible existence of a bottom-up effect of domain-specific self-efficacy on general self-efficacy. To provide more conclusive evidence on this debate, Grether et al. (2018) proposed a number of options, such as including potential confounders like personality factors to rule out alternative explanations, or setting up experimental studies to extend the ecological validity of research.

The effects of both general and reading self-efficacy on reading proficiency are somewhat counter-intuitive. Contrary to expectations, a negative association between general self-efficacy and reading proficiency is found. Yet the coefficient is so small as to indicate no substantive relationship. This might be due to the generality of this measure, whose effect might be transmitted through domain-specific pathways. One serial pathway is through reading self-efficacy and reading enjoyment, which then relates to adolescents’ reading proficiency. In addition, although this study initially hypothesized that reading self-efficacy is related to reading proficiency, the result suggests an insignificant relationship. This runs counter to previous studies documenting a positive effect of reading self-efficacy on reading proficiency (Smith et al., 2012; Peura et al., 2019a; Ma et al., 2021). One explanation might be that perceived competence is different from actual competence. Adolescents who perceive themselves as highly proficient readers might have inflated self-perceptions of their reading ability. As recent studies suggest (Carroll and Fox, 2017), reading self-efficacy does not necessarily predict higher reading proficiency. It is also plausible, though, that the use of only three items for measuring reading self-efficacy fails to meaningfully capture individual variations. Again, the use of secondary data precludes us from making modifications.

## The Mediation of Mastery Goal Orientation and Reading Enjoyment

Some of the mechanisms underlying the relationship between parental support and adolescents’ reading proficiency are consistent with what previous studies have found. For example, the positive relationship between mastery goal orientation and reading enjoyment is backed up by empirical evidence in the literature. Song et al. (2015) observed that adolescents who receive positive messages from social agents are likely to adopt mastery goals, which are associated with positive emotional responses. Donovan et al. (2018) also reported that children with mastery goals tend to have greater task engagement since they focus on knowledge acquisition and the learning process. In a meta-analysis, Huang (2011) summarized the relationship between achievement goals and achievement emotions such as enjoyment and interest. He found that mastery-approach goals have a consistently positive relationship with positive emotions.

Also, this study indicates that reading enjoyment serves as a mediator for the effect of reading self-efficacy. This finding is consistent with that of Pekrun (2006), who conceptualized perceived competence as an antecedent of academic emotions such as happiness and enjoyment. It is also consistent with empirical evidence that supports a positive link between reading self-efficacy and reading engagement (e.g., Ma et al., 2021). One reason might be that as children become more proficient in reading, they can better handle different reading materials and overcome potential challenges. As a result, it is easier for them to enjoy the reading process.

## Limitations and Implications

Several limitations of this study must be acknowledged. First, the cross-sectional data used in this study does not permit causal statements. To overcome this limitation, longitudinal designs might be considered in the future. Second, perceived support from parents is measured as a general construct in this study. Developing more fine-grained measures of such support in different contexts might provide more useful information about what types of support are most effective. Third, as the foregoing discussions have mentioned, some constructs in this study are measured with only a limited number of items in PISA 2018, such as parental support, mastery goal orientation, and reading self-efficacy, which may not fully capture individual variations. Such a methodological limitation might explain some of the statistically significant but substantively negligible relationships found in this study. Future studies might use properly designed and validated questionnaires to overcome this limitation.

Despite these limitations, this study has important implications for educational theory and practice. Theoretically, this study applies social cognitive theory to systematically investigate the different motivational pathways underlying the association between perceived parental support and adolescents’ reading proficiency. Although motivation has been widely studied as a key mechanism for socially regulated behaviors, few studies have examined self-efficacy, goals and values simultaneously. There is limited understanding of how different types of motivation interact with each other and how they relate to achievement at different levels of specificity. By differentiating between general and reading self-efficacy, this study also contributes to the scholarship on top-down and bottom-up theories of self-efficacy beliefs, indicating that a top-down perspective is more appropriate in the reading context. From a practical standpoint, this study highlights the importance of parents’ continued support during adolescence. For example, although only a weak relationship is observed between perceived support and reading proficiency, the indirect pathways suggest that positive messages from parents can work in a subtle way by boosting adolescents’ motivation, increasing their general sense of confidence and propelling them to adopt mastery goals. Such motivational processes can translate into greater task engagement (reading enjoyment) and ultimately lead to better achievement. Therefore, even if parents no longer provide explicit instruction during this period, their encouragement can implicitly serve as an impetus for adolescents to form their identity and develop a love of learning, which carries substantive benefits for achievement as well.

Future studies could further advance the field in three ways. First, it would be interesting to investigate grade differences in adolescents’ reading proficiency, which could shed light on the growth trajectories or developmental patterns of adolescents’ learning. Second, gender differences in reading comprehension have been well-documented in the literature. Whether the pathways identified in this study differ across gender should be addressed as well. Since this study is primarily concerned with examining the motivational pathways, student grade and gender are controlled rather than investigated in their own right. Addressing these issues could contribute to a better understanding of the heterogeneity and boundary conditions of our findings across different groups. Finally, future studies might also investigate whether the relationships identified in this study hold across different cultural contexts. Even though the sample used in this study is relatively large, the generalizability of our findings is limited since only the China sample is used. China is a country where parents hold high expectations about their children’s academic performance due to intense social pressure and many even see themselves as directly responsible for their children’ learning. It is worthwhile to explore the tenability of these relationships in cultures where parents hold different parenting beliefs and investigate how adolescents perceive parents’ involvement in their learning by drawing on data from more PISA participants.

## Data Availability Statement

The original contributions presented in the study are included in the article/Supplementary Material, further inquiries can be directed to the corresponding author.

## Ethics Statement

The studies involving human participants were reviewed and approved by The Ethics Committee of the School of International Studies, Zhejiang University. Written informed consent for participation was not required for this study in accordance with the national legislation and the institutional requirements.

## Author Contributions

XC: design of the study, data analysis and interpretation, and paper writing. JH: design of the study, interpretation of data for the work, paper writing, paper revision, and supervision. All authors contributed to the article and approved the submitted version.

## Funding

This work was supported by the National Social Science Fund of China (grant number 21BYY024).

## Conflict of Interest

The authors declare that the research was conducted in the absence of any commercial or financial relationships that could be construed as a potential conflict of interest.

## Publisher’s Note

All claims expressed in this article are solely those of the authors and do not necessarily represent those of their affiliated organizations, or those of the publisher, the editors and the reviewers. Any product that may be evaluated in this article, or claim that may be made by its manufacturer, is not guaranteed or endorsed by the publisher.

## APPENDIX A: Questionnaire Items Corresponding To the Main Variables.

**Table:**

Construct	Item ID	Item wording
Support	ST123Q02NA	My parents support my educational efforts and achievements.
	ST123Q03NA	My parents support me when I am facing difficulties at school.
	ST123Q04NA	My parents encourage me to be confident.
G-Efficacy	ST188Q01HA	I usually manage one way or another.
	ST188Q02HA	I feel proud that I have accomplished things.
	ST188Q03HA	I feel that I can handle many things at a time.
	ST188Q06HA	My belief in myself gets me through hard times.
	ST188Q07HA	When I’m in a difficult situation, I can usually find my way out of it.
Mastery	ST208Q01HA	My goal is to learn as much as possible.
	ST208Q02HA	My goal is to completely master the material presented in my classes.
	ST208Q04HA	My goal is to understand the content of my classes as thoroughly as possible.
R-Efficacy	ST161Q01HA	I am a good reader.
	ST161Q02HA	I am able to understand difficult texts.
	ST161Q03HA	I read fluently.
Enjoyment	ST160Q01IA	I read only if I have to.
	ST160Q02IA	Reading is one of my favourite hobbies.
	ST160Q03IA	I like talking about books with other people.
	ST160Q04IA	For me, reading is a waste of time.
	ST160Q05IA	I read only to get information that I need.

### APPENDIX B: Bivariate Correlations Among All the Variables.

**Table:**

	1	2	3	4	5	6	7	8	9	10	11
Support	1										
G-Efficacy	0.314^**^	1									
Mastery	0.294^**^	0.362^**^	1								
R-Efficacy	0.233^**^	0.448^**^	0.265^**^	1							
Enjoyment	0.248^**^	0.283^**^	0.243^**^	0.595^**^	1						
Read^*^	0.175^**^	0.038^**^	0.137^**^	0.195^**^	0.310^**^	1					
Gender	−0.028^**^	0.110^**^	−0.024^**^	−0.011	−0.145^**^	−0.085^**^	1				
ESCS	0.173^**^	0.187^**^	0.161^**^	0.287^**^	0.201^**^	0.384^**^	−0.020^*^	1			
Type	0.026^**^	0.009	−0.010	0.019^*^	0.016	0.031^**^	0.039^**^	0.080^**^	1		
Size	0.006	0.000	−0.030^**^	−0.011	0.008	0.066^**^	0.001	0.007	0.109^**^	1	
Ratio	−0.041^**^	−0.040^**^	−0.064^**^	−0.057^**^	−0.040^**^	−0.093^**^	0.019^*^	−0.133^**^	0.284^**^	0.582^**^	1

Note: Only the first plausible value of reading proficiency is used for illustration purposes.

* Correlation is significant at the. 0.05 level (2-tailed);

** Correlation is significant at the. 0.01 level (2-tailed).

### APPENDIX C: Effect of the Control Variables.

**Table:**

	G-Efficacy	Mastery	R-Efficacy	Enjoyment	Read
Gender	−0.0243 (0.017)	0.029 (0.018)	0.096 (0.016)	0.269 (0.015)	0.013 (0.016)
ESCS	0.139 (0.010)	0.113 (0.010)	0.199 (0.009)	0.014 (0.009)	0.104 (0.010)
Type					−0.157 (0.080)
Size					0.183 (0.039)
Ratio					−0.184 (0.054)

Note: Gender and ESCS are used as controls for all the endogenous variables, while school type, school size, and student–teacher ratio are used as controls for adolescents’ reading proficiency only. These effects are statistically significant at the 0.001 level. No asterisks are provided to save space.
